# Entrepreneurship Awareness and Intention After a Training Program in Nursing Interns: A Pretest–Posttest Study

**DOI:** 10.3390/nursrep16090314

**Published:** 2026-09-02

**Authors:** Eman Ramadan Abdalfadeel, Eman Kamel Hossny, Ahmed Zinhom Elkady, Mohannad Aldiqs

**Affiliations:** 1 Primary Care Nursing Department, Faculty of Nursing, Al-Ahliyya Amman University, Amman 19328, Jordan; m.aldiqs@ammanu.edu.jo; 2 Nursing Administration Department, Faculty of Nursing, Cairo University, Giza 12211, Egypt; 3 Community and Mental Health Nursing Department, Faculty of Nursing, Zarqa University, Zarqa 13132, Jordan; 4 Nursing Administration Department, Faculty of Nursing, Assiut University, Assiut 71515, Egypt; 5 Nursing Department, North Private College of Nursing, Arar 73311, Northern Borders Region, Saudi Arabia; ahmadzinhom@nec.edu.sa

**Keywords:** entrepreneurship, entrepreneurship awareness, entrepreneurial intention, entrepreneurship education, nursing interns, nursing education, training program

## Abstract

**Background:** Entrepreneurship has become an essential competency for healthcare professionals because it promotes innovation, autonomy, and the development of sustainable healthcare services. Although entrepreneurship education has demonstrated positive effects on students’ entrepreneurial knowledge and competencies, evidence regarding its effectiveness among nursing interns remains limited. This study examined changes in nursing interns’ entrepreneurship awareness, entrepreneurial intention, and entrepreneurial characteristics following participation in a structured entrepreneurship training program. **Methods:** A quasi-experimental one-group pretest–posttest design with a two-month follow-up assessment was conducted among 168 nursing interns at the Faculty of Nursing, Cairo University. Participants completed three researcher-developed instruments assessing entrepreneurship awareness, entrepreneurial intention, and entrepreneurial characteristics. The intervention consisted of a structured entrepreneurship training program delivered through interactive educational strategies, including lectures, group discussions, brainstorming, case-based learning, and practical activities. Data were analyzed using repeated-measures analysis of variance (ANOVA) and Pearson correlation coefficients. Bonferroni-corrected pairwise comparisons were performed following significant overall repeated-measures effects. **Results:** Entrepreneurship awareness increased significantly across the three assessment periods, with scores increasing from 6.41 ± 3.16 at baseline to 16.99 ± 3.45 immediately after the intervention and remaining significantly higher at the two-month follow-up (14.42 ± 4.01; *p* < 0.001). Significant improvements were also observed in overall entrepreneurial characteristics (*F* = 158.08, *p* < 0.001, $\eta_{p}^{2}$ = 0.49), particularly perseverance, demand for efficiency and quality, commitment to work, information seeking, goal setting, and planning. In contrast, entrepreneurial intention did not change significantly across the three assessment periods (*F* = 1.22, *p* = 0.30, $\eta_{p}^{2}$ = 0.01). Entrepreneurship awareness was weakly and positively correlated with entrepreneurial intention across the assessment periods (*r* = 0.165–0.263, *p* < 0.05). **Conclusions:** Participation in the entrepreneurship training program was associated with significant improvements in entrepreneurship awareness and entrepreneurial characteristics among nursing interns, whereas entrepreneurial intention did not demonstrate a statistically significant change. Integrating entrepreneurship education into undergraduate nursing curricula may strengthen innovation-related competencies and prepare future nurses to develop sustainable healthcare initiatives. Future controlled longitudinal studies should investigate the psychological and contextual factors that may influence the development of entrepreneurial intention.

## 1. Introduction

Entrepreneurship is increasingly recognized as an important driver of economic development, innovation, employment creation, and sustainable growth across multiple sectors, including healthcare. Within healthcare systems, entrepreneurial activities can contribute to the development of innovative healthcare services, improved organizational efficiency, and sustainable solutions to increasingly complex clinical and managerial challenges. These contributions are particularly important as healthcare systems face rising demands related to population growth, technological advancement, workforce shortages, changing patient expectations, and limited resources [1].

Nurses represent the largest professional workforce within many healthcare systems and are uniquely positioned to identify unmet patients and community needs because of their extensive clinical expertise and continuous interaction with patients, families, and healthcare teams. Nursing entrepreneurship extends beyond establishing independent businesses and may include developing innovative healthcare services, introducing nurse-led care models, implementing evidence-based innovations, improving patient education, designing health-promotion initiatives, and creating practical solutions to clinical and organizational problems. Entrepreneurial competencies have also been associated with professional autonomy, career development, leadership capacity, and improved quality and sustainability of healthcare services [2].

Entrepreneurship awareness represents an important foundation for entrepreneurial development because it reflects individuals’ understanding of entrepreneurial concepts, opportunity recognition, innovation, and the processes required to transform an idea into a service or business. Among nursing interns, developing entrepreneurship awareness may broaden perceptions of professional and career opportunities beyond conventional clinical employment and encourage them to view clinical and community health problems as opportunities for innovation. Nevertheless, entrepreneurship, innovation, financial management, marketing, and business planning remain insufficiently integrated into many nursing curricula, which may limit nursing students’ preparedness to identify and pursue healthcare-related entrepreneurial opportunities [3].

Recent evidence has emphasized the growing importance of entrepreneurship education in nursing. Studies have shown that entrepreneurship education and training can improve nursing students’ innovation skills, entrepreneurial knowledge, attitudes, competencies, and motivation. Innovation-focused and experiential courses have also been associated with improvements in creative thinking and entrepreneurial skills among nursing students. In Egypt, studies among nursing students and interns have reported positive effects of structured entrepreneurship education on innovation potential, entrepreneurship knowledge, attitudes, skills, motivation, and entrepreneurial readiness. However, entrepreneurship education remains inconsistently integrated into nursing curricula, and evidence regarding its effect on entrepreneurial intention among nursing interns remains limited, highlighting the need to strengthen educational and organizational factors that support nurses’ professional development and performance [4].

Entrepreneurial intention refers to an individual’s conscious willingness and commitment to engage in entrepreneurial activity and is considered an important proximal predictor of entrepreneurial behavior. Previous studies among healthcare and nursing students have reported that personal characteristics, entrepreneurial competencies, managerial readiness, and educational experiences may influence entrepreneurial intentions. Entrepreneurial intention is also affected by social, psychological, and contextual factors, including students’ perceptions of the feasibility and desirability of entrepreneurship and the level of support available within their professional and social environments [5].

Nursing interns represent a critical transitional group moving from academic preparation to professional practice, making this stage particularly suitable for developing entrepreneurial competencies that may influence future career pathways. However, evidence evaluating structured entrepreneurship training among nursing interns remains limited, particularly in developing countries. Furthermore, few intervention studies have simultaneously examined changes in entrepreneurship awareness and entrepreneurial intention following structured educational programs, despite the theoretical relationship between these constructs proposed by the Theory of Planned Behavior [6].

Despite growing international and regional interest in nursing entrepreneurship education, empirical evidence evaluating structured entrepreneurship training among nursing interns remains limited, particularly in the Egyptian nursing education context. Therefore, this study aimed to evaluate the effect of a structured entrepreneurship training program on nursing interns’ entrepreneurship awareness, entrepreneurial intention, and entrepreneurial characteristics. The findings may support the integration of entrepreneurship education into undergraduate nursing curricula in Egypt and contribute to preparing innovative nurse leaders capable of developing sustainable healthcare solutions [7].

## 2. Theoretical Framework

### 2.1. Theory of Planned Behavior

The Theory of Planned Behavior provides the primary theoretical framework for understanding entrepreneurial intention in the present study. The theory proposes that intention to perform a behavior is mainly determined by three constructs: attitude toward the behavior, subjective norms, and perceived behavioral control [8].

Attitude toward entrepreneurship refers to the extent to which nursing interns evaluate entrepreneurial activity positively or negatively. A positive attitude may develop when participants perceive entrepreneurship as beneficial, achievable, professionally meaningful, and capable of improving healthcare delivery. The training program addressed this construct through lectures, discussions, and healthcare-related examples demonstrating the professional, social, and economic value of nurse-led innovation and entrepreneurship [9].

Subjective norms refer to participants’ perceptions of whether important individuals or groups, such as family members, peers, educators, professional colleagues, and healthcare organizations, support or approve of their engagement in entrepreneurship. The intervention addressed subjective norms through group discussions, collaborative activities, and examples of nurses and healthcare professionals who had developed innovative services or entrepreneurial initiatives. These activities were intended to demonstrate that entrepreneurship is compatible with nursing identity and professional practice [10].

Perceived behavioral control refers to participants’ perceived ability and confidence to perform entrepreneurial activities. In the context of nursing entrepreneurship, this includes the ability to identify opportunities, generate ideas, develop a business or service plan, identify resources, manage risks, solve problems, and initiate a healthcare-related project. The training program addressed perceived behavioral control through brainstorming, practical exercises, opportunity-recognition activities, goal setting, planning, case discussions, and problem-solving tasks [11].

According to the Theory of Planned Behavior, attitudes, subjective norms, and perceived behavioral control contribute to the formation of entrepreneurial intention. Therefore, the training program was expected to enhance entrepreneurial intention by increasing participants’ awareness of healthcare opportunities, improving their evaluations of entrepreneurship, strengthening their perceived capabilities, and increasing their perception of social and professional support [12].

### 2.2. Entrepreneurial Event Model

The Entrepreneurial Event Model provides a complementary explanation of entrepreneurial intention. The model proposes that entrepreneurial intention is influenced by perceived desirability, perceived feasibility, and an individual’s propensity to act [13].

Perceived desirability refers to the degree to which an individual views entrepreneurship as attractive or desirable. The training program was expected to increase desirability by presenting the potential professional, social, and economic benefits of healthcare entrepreneurship and by illustrating how nurse-led initiatives can address unmet patient and community needs [14].

Perceived feasibility refers to the extent to which an individual believes that entrepreneurship is possible and achievable. Practical learning activities, examples, guided exercises, and discussions of healthcare opportunities were intended to increase participants’ perceptions that they could develop and implement entrepreneurial ideas [15].

Propensity to act refers to an individual’s tendency to initiate action when an opportunity is identified. Brainstorming, goal setting, planning, and problem-solving activities were included to encourage participants to move beyond awareness and consider practical steps toward developing healthcare-related entrepreneurial initiatives [16]. The Entrepreneurial Event Model complements the Theory of Planned Behavior by emphasizing the perceived desirability and feasibility of entrepreneurship and the transition from recognizing an opportunity to intending to act [17].

### 2.3. Human Capital Theory

Human Capital Theory provides an additional framework for explaining the potential effect of entrepreneurship education. The theory proposes that education and training enhance individuals’ knowledge, skills, abilities, and productivity, thereby increasing their capacity to identify and pursue professional and economic opportunities [18].

In nursing education, entrepreneurship training can be viewed as an investment in human capital. The program was designed to develop knowledge and skills related to opportunity recognition, creativity, innovation, communication, leadership, planning, problem-solving, resource identification, and risk management. These competencies may help nursing interns recognize healthcare opportunities and increase their confidence in developing entrepreneurial solutions [19].

Human Capital Theory also explains the expected relationship between entrepreneurship education and entrepreneurial characteristics. By providing structured learning experiences and opportunities for practice, the intervention was expected to strengthen characteristics such as opportunity seeking, perseverance, demand for efficiency and quality, commitment to work, information seeking, goal setting, planning, persuasion and networking, risk-taking, and self-confidence [20].

### 2.4. Application of the Theoretical Framework

The three theoretical perspectives provide complementary explanations for the expected effects of the intervention. The Theory of Planned Behavior explains how attitudes, subjective norms, and perceived behavioral control may influence entrepreneurial intention. The Entrepreneurial Event Model explains how training may increase the perceived desirability and feasibility of entrepreneurship and encourage a propensity to act. Human Capital Theory explains how education and training may develop the knowledge, skills, and entrepreneurial characteristics required to recognize and pursue healthcare opportunities [21].

Accordingly, the intervention was expected to increase entrepreneurship awareness by improving participants’ knowledge of entrepreneurial concepts, innovation, opportunity recognition, and healthcare business opportunities. It was also expected to improve attitudes toward entrepreneurship, strengthen perceived behavioral control, enhance subjective norms, and develop entrepreneurial characteristics, including opportunity seeking, perseverance, risk-taking, demand for efficiency and quality, commitment to work, information seeking, goal setting, planning, persuasion and networking, and self-confidence [22].

Based on these theoretical assumptions, entrepreneurship awareness, entrepreneurial characteristics, and entrepreneurial intention were selected as the main outcomes of the study. The training program was expected to improve these outcomes immediately after implementation and maintain the improvement at the two-month follow-up assessment [23].

#### Research Hypotheses

**H1.**  *The entrepreneurship training program significantly improves nursing interns’ entrepreneurship awareness*.

**H2.**  *The entrepreneurship training program significantly improves nursing interns’ entrepreneurial intention*.

## 3. Materials and Methods

### 3.1. Study Design

A quasi-experimental, one-group pretest–posttest design with a two-month follow-up assessment was used to examine changes in nursing interns’ entrepreneurship awareness and entrepreneurial intention following an entrepreneurship training program. Participants completed the study questionnaires at three points: before the intervention (pretest), immediately after completing the program (posttest), and two months after the intervention (follow-up). This design allowed the assessment of immediate and short-term changes associated with the educational intervention.

However, because the study did not include a control group or randomization, the findings cannot establish a causal relationship between the training program and the observed outcomes. Improvements may have been influenced by alternative explanations, including testing effects, regression to the mean, maturation, or other concurrent learning experiences. Therefore, the results were interpreted as changes temporally associated with participation in the program rather than definitive evidence of intervention effectiveness. The study is reported in accordance with the Transparent Reporting of Evaluations with Nonrandomized Designs (TREND) statement.

### 3.2. Study Setting and Participants

The study was conducted at the Faculty of Nursing, Cairo University, Egypt, during the nursing internship training year. Nursing interns were selected because they represent the transitional stage between undergraduate education and professional nursing practice, making this period particularly appropriate for developing entrepreneurial competencies.

A convenience sampling technique was adopted. All nursing interns who were available during the study period and met the eligibility criteria were invited to participate voluntarily.

The inclusion criteria were:

Enrollment in the nursing internship program at Cairo University; willingness to participate in the study; and attendance throughout the entrepreneurship training program. Interns who declined participation or failed to complete the study assessments were excluded from the final analysis.

### 3.3. Sample Size

A priori power analysis was conducted using G*Power 3.1 [24] (Faul et al., 2007) to determine the minimum sample size required for a one-way repeated-measures ANOVA with a within-subjects factor of time (three levels: pretest, posttest, and two-month follow-up). The analysis was specified with α = 0.05, power (1 − β) = 0.95, and a medium effect size of f = 0.25, consistent with conventional benchmarks for ANOVA [25].

The design assumed three repeated measurements (k = 3; df_num = 2), an average correlation among repeated measures of *r* = 0.50, and a Greenhouse–Geisser nonsphericity correction of ε = 1.00 (sphericity assumed). Under these parameters, the analysis yielded a non-centrality parameter λ = 16.125, a critical *F* value of 3.106, numerator degrees of freedom *df*_1_ = 2, and denominator degrees of freedom *df*_2_ = 84. The projected total sample size was N = 43 participants, which provided an actual power of 0.951. Accordingly, a minimum of 43 participants were required to detect the hypothesized within-subjects effect with adequate statistical sensitivity.

Initially, 200 nursing interns were invited to participate. Of these, 168 provided written informed consent and completed the baseline assessment. All 168 participants subsequently completed the posttest immediately after the intervention and the two-month follow-up assessment; therefore, no loss to follow-up occurred and the final analytical sample consisted of 168 participants at each assessment point. Because complete data were available for all participants across the three assessment periods, no imputation or other statistical methods for handling missing outcome data were required. The analyses were conducted using the same 168 participants at baseline, posttest, and follow-up.

Eligible nursing interns were informed about the study during internship orientation sessions. Participation was entirely voluntary, with no financial or academic incentives provided. Written informed consent was obtained from all participants before enrollment.

### 3.4. Intervention Development

Prior to implementing the study, official approval was obtained from the Vice Dean for Education and Student Affairs and the Head of the Nursing Administration Department, Faculty of Nursing, Cairo University. Following administrative approval, the study instruments were developed based on an extensive review of the relevant literature and were evaluated for content validity and reliability before data collection. The educational setting, training schedule, and communication channels with participants were also prepared to facilitate implementation of the intervention.

#### 3.4.1. Entrepreneurship Training Program

The entrepreneurship training program was developed according to the baseline educational needs identified during the pre-intervention assessment and current evidence regarding entrepreneurship education for healthcare professionals. The program was designed to improve nursing interns’ entrepreneurship awareness and promote entrepreneurial thinking within healthcare practice.

#### 3.4.2. Program Structure and Delivery

The training program consisted of six interactive sessions delivered over a period of three consecutive weeks (two sessions per week). The total instructional time was 12 h, with each session lasting 2 h. Participants were trained in a single cohort to ensure consistency in content delivery and to facilitate group dynamics. All educational sessions were held in the hospital’s main conference room.

#### 3.4.3. Educational Content

The educational content addressed the following topics:

- Concepts and principles of entrepreneurship;
- Characteristics of successful entrepreneurs;
- Entrepreneurship opportunities in nursing and healthcare;
- Innovation and opportunity recognition;
- Business planning and project development;
- Entrepreneurial challenges and risk management; and
- Nurse-led entrepreneurial initiatives and success stories.

#### 3.4.4. Implementation

Because this was a one-group quasi-experimental study, all eligible participants received the intervention, and no allocation or randomization procedures were performed. The program employed learner-centered educational strategies, including interactive lectures, group discussions, brainstorming sessions, case-based learning, and structured feedback. These teaching methods were selected to encourage active participation, critical thinking, collaborative learning, and the application of entrepreneurial concepts to nursing practice. All educational sessions were delivered by the principal investigator using standardized educational materials to ensure consistency throughout the intervention. Attendance was monitored throughout the program to maximize intervention fidelity. Blinding was not feasible because participants and investigators were aware of the educational intervention. All enrolled participants completed the intervention sessions and outcome assessments.

### 3.5. Data Collection Procedure

Data collection was conducted at three measurement points: pre-intervention, immediately post-intervention, and two-month follow-up. The intervention was structured according to the Theory of Planned Behavior (TPB), which proposes that entrepreneurial intention is influenced by attitudes toward behavior, subjective norms, and perceived behavioral control. Accordingly, the training activities were designed to increase participants’ awareness of healthcare entrepreneurship, develop favorable attitudes toward entrepreneurial activities, strengthen perceived capability and control over initiating entrepreneurial projects, and enhance perceived social support for pursuing entrepreneurship.

#### 3.5.1. Pre-Intervention Assessment

At baseline, the investigator explained the study purpose, procedures, voluntary nature of participation, and confidentiality measures. Written informed consent was obtained before enrollment. Communication methods and the training schedule were agreed upon with the participants before data collection.

Participants then completed the Entrepreneurship Awareness Tool and the Nursing Interns’ Entrepreneurial Intention Questionnaire under the investigator’s supervision. In addition to measuring entrepreneurship awareness and entrepreneurial intention, the baseline assessment was used to identify participants’ perceptions relevant to the TPB constructs, including their attitudes toward healthcare entrepreneurship, perceived behavioral control, and perceived subjective norms. Questionnaire completion required approximately 15 min.

#### 3.5.2. Intervention Implementation

After the baseline assessment, participants attended a structured entrepreneurship training program. The program was designed to influence entrepreneurial intention through the three primary TPB mechanisms.

The first component targeted attitudes toward entrepreneurship through lectures and discussions on the importance, benefits, feasibility, and potential contribution of healthcare entrepreneurship to nursing practice and patient care. Examples of successful healthcare businesses and nurse-led innovations were presented to help participants develop more favorable evaluations of entrepreneurial activities.

The second component targeted perceived behavioral control through brainstorming activities, practical examples, and guided exercises related to opportunity identification, idea generation, business planning, resource identification, problem-solving, goal setting, and managing potential risks. These activities were intended to increase participants’ confidence in their ability to recognize and pursue healthcare-related entrepreneurial opportunities.

The third component targeted subjective norms through interactive discussions concerning the role of nurses, educators, healthcare organizations, families, and professional colleagues in supporting entrepreneurial initiatives. Participants were encouraged to discuss perceived social expectations, possible sources of support, and barriers that might influence their decision to engage in entrepreneurship.

The training used active-learning strategies, including lectures, interactive discussions, brainstorming exercises, group activities, case examples, and practical healthcare-related applications. Through these activities, the program was expected to increase entrepreneurship awareness and entrepreneurial characteristics while strengthening favorable attitudes, perceived behavioral control, and perceived social support, thereby supporting the development of entrepreneurial intention in accordance with the TPB framework.

#### 3.5.3. Post-Intervention Evaluation

Immediately after completion of the training program, participants completed the same study instruments to assess the immediate effects of the intervention on entrepreneurship awareness, entrepreneurial intention, and entrepreneurial characteristics. The post-intervention assessment also examined whether the training had strengthened the TPB-related mechanisms expected to contribute to entrepreneurial intention, particularly favorable attitudes toward entrepreneurship, perceived behavioral control, and subjective norms.

A follow-up assessment was conducted two months after completion of the program using the same instruments. The follow-up assessment was intended to determine whether improvements in entrepreneurship awareness, entrepreneurial characteristics, and entrepreneurial intention were retained over time. It also assessed whether the anticipated effects of the intervention on participants’ attitudes, perceived behavioral control, and subjective norms were maintained beyond the immediate post-intervention period.

### 3.6. Study Instruments

Data were collected using three researcher-developed instruments based on an extensive review of the relevant entrepreneurship, entrepreneurship education, healthcare, and nursing education literature. The instruments were designed to assess nursing interns’ entrepreneurship awareness, entrepreneurial intention, and entrepreneurial characteristics.

#### 3.6.1. Entrepreneurship Awareness Tool

The Entrepreneurship Awareness Tool was developed by researchers based on the relevant entrepreneurship literature and the work of Krueger and Carsrud [26]. It consisted of two sections. Section I: Demographic and background characteristics: This section collected participants’ demographic and background information, including, gender, marital status, place of residence, previous self-employment experience, previous entrepreneurship-related training, and whether a family member or close relative was self-employed. Section II: Entrepreneurship Awareness Knowledge Test: This section consisted of 20 multiple-choice items designed to assess nursing interns’ foundational knowledge and awareness of entrepreneurship, business opportunities, and innovation.

The items covered basic entrepreneurship concepts, recognition of business and market opportunities, innovation, and the application of entrepreneurship within the healthcare sector. Each item had one correct answer. A score of one was assigned for each correct answer, whereas an incorrect answer was assigned a score of zero. The total score ranged from 0 to 20, with higher scores indicating a higher level of entrepreneurship awareness. An example item assesses knowledge of how business ideas are generated (e.g., experience, daydreams, or imagination). The knowledge test assessed basic cognitive awareness of healthcare-related entrepreneurial opportunities rather than advanced entrepreneurial competence, practical business skills, or actual entrepreneurial performance.

#### 3.6.2. Nursing Interns’ Entrepreneurial Intention Questionnaire

The Nursing Interns’ Entrepreneurial Intention Questionnaire was developed by researchers based on the conceptual framework proposed by Crant [27] and the relevant literature on entrepreneurial intention. The questionnaire consisted of seven items assessing nursing interns’ willingness and future intention to engage in entrepreneurial activities within the healthcare sector. The items addressed the participants’ intention to pursue healthcare-related business activities, willingness to establish or participate in entrepreneurial projects, and readiness to consider entrepreneurship as a future career or professional activity. Responses were recorded using a three-point Likert scale as follows: 1 = disagree, 2 = neutral, and 3 = agree. Individual item scores were summed up to obtain a total score ranging from 7 to 21. Higher total scores indicated stronger entrepreneurial intention. An example item includes: “*I have a strong intention to start a business someday.*” A three-point response format was selected to reduce response burden and cognitive fatigue among nursing interns during their clinical training. It was also intended to encourage completion and facilitate clear responses. However, the limited number of response categories may reduce response variability and is acknowledged as a limitation of the instrument.

#### 3.6.3. Entrepreneurial Characteristics Scale

The Entrepreneurial Characteristics Scale was developed by researchers based on the relevant entrepreneurship literature and the entrepreneurial characteristics framework described by McClelland [28]. The scale was designed to assess entrepreneurial characteristics relevant to nursing interns and healthcare entrepreneurship. The scale assessed 10 entrepreneurial characteristics across 25 items: opportunity seeking (2 items), perseverance (2 items), risk-taking (2 items), demand for efficiency and quality (2 items), commitment to work (2 items), information seeking (3 items), goal setting (2 items), planning (3 items), persuasion and networking (3 items), and self-confidence (4 items). Each characteristic was assessed through a group of items. Scores for the items related to each characteristic were summed to obtain the corresponding subscale score.

A total entrepreneurial characteristics score was calculated by summing the scores across all dimensions, with total scores spanning from 25 to 75. Higher scores indicated stronger entrepreneurial characteristics. An example item from the opportunity seeking subscale is: “I act upon the opportunities and problems present.” The scale assessed participants’ self-reported entrepreneurial characteristics and tendencies rather than objectively observed entrepreneurial behavior, business ownership, or actual entrepreneurial performance. Accordingly, the scores reflected participants perceived entrepreneurial dispositions at each assessment point.

### 3.7. Validity and Reliability

Content validity of all study instruments was established by a panel of five experts in nursing administration and entrepreneurship education, who evaluated relevance, clarity, comprehensiveness, readability, and appropriateness. Instruments were revised according to expert recommendations, yielding scale content validity indices (S-CVI) above 0.80 for all three tools.

Reliability was assessed using appropriate coefficients based on item response formats. The Entrepreneurship Awareness Tool (dichotomously scored: 0 = incorrect, 1 = correct) demonstrated very high internal consistency (KR-20 = 0.91). The Nursing Interns’ Entrepreneurial Intention Questionnaire (three-point scale) showed excellent internal consistency (Cronbach’s α = 0.94). The Entrepreneurial Characteristics Scale (25 items, three-point Likert: 1 = disagree, 2 = neutral, 3 = agree) exhibited excellent internal consistency (α = 0.93), with subscale alphas ranging from 0.78 to 0.89. Split-half reliability coefficients for all instruments were satisfactory to good (*r* > 0.80).

Construct validity of the Entrepreneurial Characteristics Scale was confirmed through one-factor confirmatory factor analysis (CFA) using DWLS estimation, which demonstrated good model fit: *χ*^2^(275) = 368.54, *p* < 0.001; CFI = 0.970; TLI = 0.967; RMSEA = 0.045 (90% CI [0.032, 0.057]); SRMR = 0.081; GFI = 0.977. All factor loadings were significant (*p* < 0.001), supporting convergent validity. Internal consistency was excellent (McDonald’s ω = 0.957; Cronbach’s α = 0.919).

Because the Entrepreneurial Characteristics Scale uses a 3-point ordinal response format, standard Cronbach’s alpha (based on Pearson correlations) may underestimate true reliability and is sensitive to range restriction. To address this, we re-estimated internal consistency using ordinal alpha and ordinal omega, computed from the polychoric correlation matrix, as recommended for Likert-type items with few response categories. The resulting coefficients remained excellent (ordinal α = 0.919; ordinal ω = 0.957), confirming strong internal consistency under assumptions more appropriate for ordinal data.

The entrepreneurship training program was independently reviewed by three academic experts for relevance, scientific accuracy, educational content, clarity, and alignment with study objectives. Minor modifications were incorporated based on their recommendations before implementation.

It should be noted that item-level psychometric analyses (e.g., item difficulty and discrimination indices) were not performed for the Entrepreneurship Awareness Knowledge Test. Future research should undertake comprehensive psychometric evaluation, including item-level analysis and construct validation, to further establish its measurement properties.

### 3.8. Statistical Analysis

Data were coded, entered, and analyzed using IBM SPSS version 30 (IBM Corp., Armonk, NY, USA), with descriptive statistics (frequencies, percentages, means, and standard deviations) used to summarize participants’ demographic characteristics and study variables, while repeated-measures analysis of variance (ANOVA) was performed to evaluate changes in entrepreneurship awareness, entrepreneurial intention, and entrepreneurial characteristics across the three assessment points (pretest, posttest, and two-month follow-up), with the within-subjects factor being time (three levels). Prior to the main analyses, assumption testing was conducted: the Shapiro–Wilk test confirmed normality of residuals across all conditions (all p>0.05).

Mauchly’s test of sphericity was performed for each dependent variable, and where sphericity was violated, the Greenhouse–Geisser correction was applied to adjust degrees of freedom (e.g., for total entrepreneurial characteristics, $df_{1}=1.58,df_{2}=263.86$), while for outcomes where sphericity was met (e.g., total entrepreneurial characteristics, $\chi^{2}=2.87,df=2,p=0.238$), unadjusted results are reported; Levene’s test confirmed homogeneity of variance across all time points (p>0.05). When a significant overall repeated-measures effect was identified (p<0.05), post hoc pairwise comparisons between the three assessment points (pretest vs. posttest, pretest vs. follow-up, and posttest vs. follow-up) were performed using estimated marginal means with the Bonferroni correction to control for Type I error, with adjusted p-values and 95% confidence intervals for mean differences reported; importantly, post hoc comparisons were conducted and interpreted only when the omnibus F-test was statistically significant, and for outcomes where the omnibus test was not significant (e.g., Entrepreneurial Intention: $F\left(1.89,315.47\right)=1.22,p=0.30$).

No post hoc pairwise comparisons were performed or interpreted, consistent with established statistical guidelines requiring that post hoc tests be conditional on a significant overall ANOVA result. Effect sizes for the repeated-measures ANOVA are reported as partial eta squared ($\eta_{p}^{2}$) along with 95% confidence intervals, interpreted as small (0.01), medium (0.06), and large (0.14). Pearson’s correlation coefficient was used to examine bivariate relationships among entrepreneurship awareness, entrepreneurial intention, and entrepreneurial characteristics at each time point, with assumptions of linearity (verified via scatterplots) and absence of extreme outliers (standardized residuals >±3.29) checked prior to analysis, and correlation coefficients interpreted as weak (∣r∣<0.30), moderate (0.30≤∣r∣<0.50), and strong (∣r∣≥0.50). There was no missing data in the final dataset, as all 168 participants completed all three assessments, thus no imputation methods were required. All statistical tests were two-tailed, with statistical significance established at α=0.05, and all reported p-values are exact values unless p<0.001, in which case they are reported as such.

### 3.9. Ethical Considerations

Ethical approval for the study was originally granted by the Research Ethics Committee at the Faculty of Nursing, Cairo University, on 15 January 2020 (Approval Reference: RHDIRB2019041701), prior to the commencement of the study. A formal renewal of the ethical approval was subsequently obtained from the Research Ethics Committee under Approval Reference HUNURSERC 2025/07/52/19, which constituted the current and valid ethical approval for the implementation of the study and data collection in 2025. Data collection officially commenced on 1 October 2025, and was completed in December 2025, ensuring that all study procedures were conducted under valid and active ethical approval. Administrative approval was also obtained from the Vice Dean for Education and Student Affairs and the Head of the Nursing Administration Department prior to initiating the study.

All participants received written and verbal information describing the study objectives, procedures, voluntary nature of participation, and confidentiality of collected data. Written informed consent was obtained from all participants before enrollment.

Participants were informed of their right to withdraw from the study at any stage without academic or personal consequences. All collected data were treated confidentially, anonymized before analysis, and used exclusively for research purposes.

## 4. Results

### 4.1. Participants’ Characteristics

A total of 168 nursing interns completed the baseline assessment. Most participants were female (60.1%), single (87.5%), and residing in rural areas (57.1%). Slightly more than half (53.6%) reported no previous self-employment experience, whereas 72.6% indicated that at least one family member or close relative was self-employed. Only 11.9% had previously attended entrepreneurship-related training programs (Table 1).

### 4.2. Changes in Entrepreneurship Awareness, Entrepreneurial Intention, and Entrepreneurial Characteristics Across the Three Assessment Periods

Entrepreneurship awareness improved significantly across the three assessment periods (*F* = 21.05, *p* < 0.001). The mean awareness score increased from 6.41 ± 3.16 at baseline to 16.99 ± 3.45 immediately after the intervention and remained significantly higher than baseline (14.42 ± 4.01) at the two-month follow-up (Table 2).

In contrast, entrepreneurial intention showed a modest increase following the intervention (17.18 ± 3.52 vs. 19.57 ± 3.57) and remained relatively stable at follow-up (18.94 ± 3.80); however, these changes did not reach statistical significance (*F* = 1.22, *p* = 0.30, $\eta_{p}^{2}$ = 0.01) (Table 2). Given the non-significant omnibus test, post hoc pairwise comparisons for entrepreneurial intention are not interpretable and are reported descriptively only.

Significant improvements were observed in several entrepreneurial characteristics following participation in the training program. Specifically, perseverance, commitment to work, demand for efficiency and quality, information seeking, goal setting, and planning demonstrated significant improvements across the three measurement periods (*p* < 0.05).

Conversely, opportunity seeking, risk-taking, persuasion and networking, and self-confidence did not significantly change over time (*p* > 0.05).

Overall entrepreneurial characteristics increased significantly after the intervention (*F* = 158.08, *p* < 0.001), with the highest mean score recorded immediately after the training program and a slight decline at the two-month follow-up while remaining above baseline values.

### 4.3. Bonferroni-Corrected Pairwise Comparisons Across Three Assessment Periods

Bonferroni-corrected pairwise comparisons (Table 3) showed significant improvements in entrepreneurship awareness from pretest to posttest (MD = −10.58, *p* < 0.001) and from pretest to follow-up (MD = −8.01, *p* < 0.001). A significant decline was observed from posttest to follow-up (MD = 2.57, *p* < 0.001), although follow-up scores remained higher than pretest scores. For entrepreneurial characteristics, a significant improvement was observed from pretest to posttest (MD = −3.19, *p* = 0.02), whereas the pretest-to-follow-up (MD = −1.89, *p* = 0.35) and posttest-to-follow-up (MD = 1.30, *p* = 0.85) comparisons were not significant. Thus, the post-intervention improvement in entrepreneurial characteristics was not statistically maintained at follow-up. For entrepreneurial intention, the omnibus repeated-measures ANOVA was not significant (*F* = 1.22, *p* = 0.30, $\eta_{p}^{2}$ = 0.01); therefore, pairwise comparisons were presented descriptively only.

### 4.4. Correlations Among Entrepreneurship Awareness, Entrepreneurial Intention, and Entrepreneurial Characteristics

As shown in Table 4, weak to moderate positive concurrent associations were observed between entrepreneurship awareness and entrepreneurial intention across all time points (*r* range = 0.165–0.263, all *p* < 0.05). Entrepreneurship awareness was moderately associated with entrepreneurial characteristics (*r* range = 0.338–0.411, all *p* < 0.001), and entrepreneurial intention showed strong concurrent associations with entrepreneurial characteristics (*r* range = 0.589–0.705, all *p* < 0.001).

## 5. Discussion

This study evaluated the observed patterns following an entrepreneurship training program regarding entrepreneurship awareness, entrepreneurial intention, and entrepreneurial characteristics among nursing interns. Participation was accompanied by substantial improvements in entrepreneurship awareness and several entrepreneurial characteristics, whereas entrepreneurial intention increased only modestly and non-significantly. These findings indicate that improvements in cognitive and behavioral aspects of entrepreneurship were observed following participation in the program, whereas entrepreneurial intention appeared less responsive over the study period. Changes in intention may involve additional psychological, experiential, and contextual influences beyond knowledge acquisition alone [29].

The demographic profile was consistent with undergraduate nursing populations, with most participants being female, single, and having limited entrepreneurial experience. Although nearly three-quarters reported having a self-employed family member, only a small proportion had received entrepreneurship-related training, highlighting a persistent curricular gap. Family entrepreneurial exposure has been recognized as an important source of entrepreneurial socialization, whereas limited curricular exposure remains a barrier to developing entrepreneurial competencies. This pattern aligns with broader trends in which entrepreneurship remains underrepresented in nursing education despite its relevance to healthcare innovation. However, some studies report that familial exposure alone can significantly predict entrepreneurial intention among health sciences students, suggesting that informal role modeling may be more influential than assumed. Systematic integration of entrepreneurial competencies into nursing curricula may therefore be valuable for preparing graduates for evolving healthcare landscapes [30].

Entrepreneurship awareness improved post-intervention and remained elevated at two-month follow-up, despite a slight decline. Similar improvements have been reported in studies demonstrating that structured entrepreneurship education enhances entrepreneurial knowledge and opportunity recognition. The learner-centered strategies used in this program, including case-based learning and real-world healthcare examples, may have contributed to active learning; however, the present study design does not allow the independent contribution of these strategies to be established. The sustained elevation in awareness at the two-month follow-up suggests that the observed gains were retained to some extent during the follow-up period, although the slight decline may reflect reduced reinforcement over time. In contrast, other studies report that knowledge gains from short-term interventions may return to baseline within weeks without opportunities for practical application. Evidence supports the role of active and experiential learning in promoting longer-term retention compared with didactic instruction [31].

Despite observed improvements in awareness, entrepreneurial intention did not increase significantly. According to the Theory of Planned Behavior, intention is shaped by attitudes, perceived behavioral control, and subjective norms rather than knowledge alone. The absence of a significant change highlights the complexity of intention formation and suggests that knowledge-based interventions alone may not adequately address attitudinal and normative factors. Conversely, several studies demonstrate that brief interventions can increase entrepreneurial intention among health sciences students when motivational components are incorporated. Research consistently indicates that perceived behavioral control, attitudes, and subjective norms are stronger predictors of intention than knowledge alone. Moreover, motivational factors and psychosocial safety climate have been identified as important factors associated with nurses’ work engagement, highlighting the potential role of motivational conditions in shaping nurses’ professional attitudes and behaviors [32,33].

Nursing interns are at an early stage of professional development and are typically focused on completing training requirements and securing employment before considering entrepreneurship. The short intervention duration may not have provided sufficient opportunities to develop self-efficacy through practical experience. Perceived barriers, including financial uncertainty and fear of failure, may also discourage entrepreneurship despite improved knowledge. Interns may therefore prioritize immediate professional stability over long-term entrepreneurial aspirations. Alternatively, some researchers argue that entrepreneurial intention can be cultivated early through inspirational role models before licensure. Similar barriers have been identified among healthcare students, including regulatory complexity and limited institutional support [34].

The observed pattern suggests that entrepreneurship awareness and entrepreneurial intention may represent related but distinct outcomes of entrepreneurship education. Although awareness improved significantly, entrepreneurial intention did not show a statistically significant change over the study period. This finding indicates that improvement in awareness does not necessarily correspond to a concurrent change in entrepreneurial intention. Integrating education with simulations, mentoring, and innovation projects could be explored in future interventions as potential approaches for strengthening experiential learning and addressing factors related to entrepreneurial intention. Awareness may therefore be considered an important educational outcome, but the present study cannot establish that it precedes or causes subsequent changes in entrepreneurial intention. Longitudinal research is needed to determine whether sustained changes in awareness are followed by changes in entrepreneurial intention over time [35].

Significant increases from pre- to post-intervention were observed in perseverance, planning, goal setting, information seeking, commitment to work, and quality orientation among nursing interns. These competencies are fundamental to entrepreneurial thinking and are also directly relevant to nursing practice. However, given the one-group pretest–posttest design, these observed gains cannot be attributed solely to the entrepreneurship training program, as they may partly reflect maturation during the internship period and repeated exposure to the same measurement instruments (testing effects). Nevertheless, planning and quality orientation remain relevant to both entrepreneurial ventures and evidence-based nursing practice [36].

No significant changes were detected in opportunity seeking, risk-taking, persuasion and networking, or self-confidence. These competencies may require longer-term experiential learning, mentoring, and supportive organizational environments. The absence of significant change may indicate that these characteristics are less responsive to short-term educational interventions, or that they are influenced by extraneous variables not controlled in this design. From a personality-based perspective, traits such as risk-taking may represent relatively stable individual differences that are not readily modified through brief educational exposure. Previous research indicates that these competencies may be better cultivated through real-world entrepreneurial experiences and mentorship than through classroom-based instruction alone [37].

Correlation analyses showed that awareness had weak associations with entrepreneurial intention, whereas entrepreneurial characteristics demonstrated stronger associations, particularly at follow-up. This pattern suggests that behavioral competencies may play a greater role in accompanying or shaping entrepreneurial intentions than knowledge alone. These associations support competency-based perspectives of entrepreneurial development, though causal inferences remain limited by the study design and potential confounding from maturation and testing threats. Alternatively, some researchers have reported stronger knowledge–intention relationships in business-focused educational programs, suggesting that these relationships may vary according to academic discipline and educational context [38]. Research grounded in the Theory of Planned Behavior further emphasizes the importance of perceived behavioral control in predicting entrepreneurial intention [39].

## 6. Conclusions

This study documented that participation in a short-term entrepreneurship training program was accompanied by observed improvements in entrepreneurship awareness and certain entrepreneurial characteristics among nursing interns, while changes in entrepreneurial intention were modest and non-significant. Rather than establishing proven intervention effectiveness, these findings indicate that awareness and behavioral tendencies showed positive shifts following the program. However, because the single-group pretest–posttest design inherently lacks a control group, these observed changes cannot be causally attributed to the training alone; they may partly reflect maturation, developmental changes during the internship period, or testing effects from repeated exposure to the measurement instruments. Furthermore, the modest, non-significant change in intention underscores that short-term education alone is insufficient to alter career trajectories. Consequently, claims regarding the practical efficacy of the training program remain speculative. The results merely highlight preliminary, descriptive patterns rather than definitive proof of educational impact, emphasizing that future research utilizing rigorous, controlled longitudinal designs is required before drawing any conclusions regarding the role of entrepreneurship training in nursing education.

## 7. Recommendations

For policymakers and healthcare authorities, it is recommended to consider pilot initiatives to integrate entrepreneurship modules into nursing education and continuing professional development, with built-in evaluation frameworks to assess effects on competencies and intentions over time. Support should be directed toward funding longitudinal and multicenter studies examining the conditions under which entrepreneurship education translates into entrepreneurial behavior and career outcomes. In addition, partnerships between universities and healthcare organizations should be encouraged to co-design and test experiential, mentorship-based entrepreneurship programs, while awaiting stronger evidence before large-scale policy adoption.

For nursing education institutions, entrepreneurship awareness and competency-building content should be incorporated into undergraduate curricula as a foundational element, with clear learning outcomes focused on knowledge, skills, and attitudes rather than immediate career intention. Experiential pedagogies, such as simulations, design thinking, and innovation projects, should be prioritized, and their incremental effects on self-efficacy and intention evaluated in future cohorts. Current findings should be treated as preliminary evidence supporting curriculum innovation, with iterative program improvements planned based on rigorous outcome evaluation.

For nursing professionals, engagement in entrepreneurship training and professional development opportunities is recommended to strengthen awareness and competencies, recognizing that changes in career intention may require sustained support and experience. Mentorship and networking opportunities with nurse entrepreneurs should be sought to build self-efficacy and explore innovation pathways within evidence-based practice. Participation in innovation projects and quality improvement initiatives is encouraged as a practical means of applying entrepreneurial skills in clinical and community settings.

For future research, multicenter, adequately powered randomized controlled trials should be conducted to examine the long-term effects of entrepreneurship education on entrepreneurial intention, behavior, and career trajectories. Mediating and moderating factors, such as entrepreneurial self-efficacy, organizational support, risk perception, and professional norms, should be investigated to explain the gap between awareness or competency gains and intention change. Furthermore, multifaceted interventions that combine education with mentorship, incubation, and real-world innovation projects should be explored to determine whether integrated approaches produce stronger effects on intention and behavior.

## 8. Practical Implications, Strengths, and Limitations

### 8.1. Practical Implications

The findings of this study have several implications for nursing education and curriculum development. First, the significant improvements in entrepreneurship awareness and selected entrepreneurial characteristics suggest that structured entrepreneurship education can effectively enhance cognitive and behavioral competencies among nursing interns. Nurse educators and curriculum planners should consider integrating entrepreneurship modules into undergraduate nursing programs, employing learner-centered strategies such as case-based learning, brainstorming, and real-world healthcare examples to facilitate active engagement. Second, the modest change in entrepreneurial intention indicates that knowledge acquisition alone is insufficient to influence career aspirations. Programs aiming to foster entrepreneurial careers should incorporate experiential components, including business simulations, mentoring, innovation laboratories, and opportunities to develop healthcare projects, alongside theoretical instruction. Third, the sustained gains in awareness at two-month follow-up suggest that even brief interventions can produce meaningful educational outcomes, though periodic reinforcement may be needed to prevent knowledge decay. Finally, the differential improvement across entrepreneurial characteristics highlights the need for tailored educational approaches; competencies such as planning and perseverance may respond well to classroom-based instruction, whereas traits like risk-taking and networking may require prolonged experiential learning and supportive organizational environments.

### 8.2. Strengths

This study has several notable strengths. First, it employed a longitudinal design with three measurement points (baseline, post-intervention, and two-month follow-up), allowing assessment of both immediate and sustained effects of the entrepreneurship training program. Second, the use of both objective measures (entrepreneurship awareness test) and self-report instruments (entrepreneurial intention and characteristics) provided a more comprehensive evaluation of outcomes than reliance on self-report alone. Third, the intervention was grounded in established educational theories and incorporated diverse learner-centered pedagogical strategies, enhancing the educational validity of the program. Fourth, the focus on nursing interns—a population underrepresented in entrepreneurship research—addresses an important gap in the literature and contributes to understanding entrepreneurial development in healthcare education. Fifth, the assessment of multiple entrepreneurial characteristics provided nuanced insights into which competencies are most amenable to educational intervention, informing future curriculum design.

### 8.3. Limitations

Several limitations should be considered when interpreting the findings. First, the one-group pretest–posttest design without a control group limits causal attribution; observed changes may have been influenced by maturation, testing effects, concurrent educational experiences, or participant expectations rather than the intervention alone. Second, convenience sampling from a single nursing faculty restricts generalizability to other institutions, countries, and broader nursing populations, particularly given the specific sample characteristics (87.5% single, 60.1% female). Third, reliance on self-report questionnaires for entrepreneurial intention and characteristics may introduce response bias, though this was partially mitigated by using an objective knowledge test for awareness. Fourth, the small percentage of participants with prior entrepreneurship training limited baseline comparisons across subgroups. Fifth, the absence of a participant flow diagram and incomplete reporting of attrition at each measurement point represent additional methodological limitations. Finally, the relatively short two-month follow-up period may not capture longer-term effects on entrepreneurial intention or behavior. Future multicenter randomized controlled trials with diverse geographical and educational settings, balanced demographic representation, and rigorous reporting standards are needed to enhance external and internal validity.

## Figures and Tables

**Table 1:** Demographic Characteristics of Participants.

Variable	Category	N	%
Gender	Male	67	39.9
	Female	101	60.1
Marital status	Single	147	87.5
	Married	21	12.5
Residence	Rural	96	57.1
	Urban	72	42.9
Previous self-employment	Yes	78	46.4
	No	90	53.6
Relative self-employed	Yes	122	72.6
	No	46	27.4
Entrepreneurship training	Yes	20	11.9
	No	148	88.1

Note: Values are presented as frequency (N) and percentage (%).

**Table 2:** Changes in Entrepreneurship Awareness.

Outcome	Pretest Mean ± SD	Posttest Mean ± SD	Follow-Up Mean ± SD	*F*	*p*	$\eta_{p}^{2}$	95% CI
Entrepreneurship awareness	6.41 ± 3.16	16.99 ± 3.45	14.42 ± 4.01	21.05	<0.01	0.11	[0.05, 0.19]
Entrepreneurial intention	17.18 ± 3.52	19.57 ± 3.57	18.94 ± 3.80	1.22	0.30	0.01	[0.00, 0.04]
Opportunity seeking	4.80 ± 1.17	5.05 ± 1.02	4.85 ± 1.26	2.15	0.12	0.01	[0.00, 0.05]
Perseverance	4.82 ± 1.04	5.34 ± 0.93	5.15 ± 1.20	9.59	<0.01	0.05	[0.01, 0.11]
Risk-taking	4.80 ± 1.13	4.89 ± 1.21	4.75 ± 1.23	0.53	0.59	0.00	[0.00, 0.03]
Demand for efficiency	4.69 ± 1.10	5.10 ± 1.03	5.07 ± 1.39	5.76	<0.01	0.03	[0.00, 0.08]
Commitment to work	4.64 ± 0.94	5.19 ± 1.11	5.14 ± 1.28	11.46	<0.01	0.06	[0.02, 0.13]
Information seeking	7.51 ± 1.55	8.10 ± 1.36	7.85 ± 1.69	5.8	<0.01	0.03	[0.00, 0.08]
Goal setting	5.16 ± 1.12	5.31 ± 0.99	4.99 ± 1.11	3.5	0.03	0.02	[0.00, 0.06]
Planning	7.50 ± 1.45	7.93 ± 1.44	7.89 ± 1.65	3.8	0.02	0.02	[0.00, 0.07]
Persuasion and networking	7.73 ± 1.47	7.81 ± 1.53	7.47 ± 1.69	1.93	0.15	0.01	[0.00, 0.05]
Self-confidence	10.42 ± 1.90	10.54 ± 1.94	10.80 ± 2.11	2.28	0.10	0.01	[0.00, 0.05]
Total entrepreneurial characteristics	62.08 ± 12.87	65.27 ± 12.55	63.97 ± 14.62	158.08	<0.01	0.49	[0.41, 0.56]

Note. *df*_1_ = 1.58, *df*_2_ = 263.86.

**Table 3:** Entrepreneurial Characteristics.

Dependent Variable	Comparison (I)	Comparison (J)	Mean Difference (I-J)	SE	*p* (Bonferroni)	95% CI
**Entrepreneurship** **Awareness**	Pretest	Posttest	−10.58 *	0.39	<0.001	[−11.50, −9.66]
	Pretest	Follow-up	−8.01 *	0.40	<0.001	[−9.07, −6.95]
	Posttest	Follow-up	2.57 *	0.40	<0.001	[1.63, 3.51]
**Entrepreneurial** **Characteristics**	Pretest	Posttest	−3.19 *	1.17	0.02	[−5.95, −0.43]
	Pretest	Follow-up	−1.89 *	1.20	0.35	[−4.70, 0.92]
	Posttest	Follow-up	1.30	1.21	0.85	[−1.54, 4.14]

Note. For entrepreneurial intention, the omnibus repeated-measures ANOVA was not statistically significant (*F* = 1.22, *p* = 0.30, $\eta_{p}^{2}$ = 0.01). Accordingly, pairwise comparisons for this outcome were not interpreted inferentially and are presented descriptively only. * *p* < 0.05.

**Table 4:** Correlations among Study Variables.

Variables	Pretest *r* (*p*)	Posttest *r* (*p*)	Follow-Up *r* (*p*)
Awareness ↔ Intention	0.165 (0.033)	0.212 (0.007)	0.263 (0.001)
Awareness ↔ Characteristics	0.411 (<0.001)	0.338 (<0.001)	0.395 (<0.001)
Intention ↔ Characteristics	0.705 (<0.001)	0.589 (<0.001)	0.625 (<0.001)

## Data Availability

Data supporting the findings of this study are available from the corresponding author upon reasonable request.

## Public Involvement Statement

There was no public involvement in any aspect of this research.

## Guidelines and Standards Statement

This manuscript was prepared and revised in accordance with the Transparent Reporting of Evaluations with Nonrandomized Designs (TREND) statement for nonrandomized intervention studies.
